# Cumulus cells accelerate oocyte aging by releasing soluble Fas Ligand in mice

**DOI:** 10.1038/srep08683

**Published:** 2015-03-03

**Authors:** Jiang Zhu, Jie Zhang, Hong Li, Tian-Yang Wang, Chuan-Xin Zhang, Ming-Jiu Luo, Jing-He Tan

**Affiliations:** 1 College of Life Science, Northeast Agricultural University, Harbin, 150030, P. R. China; 2 College of Animal Science and Veterinary Medicine, Shandong Agricultural University, Tai-an City 271018, P. R. China

## Abstract

Although previous studies have suggested that cumulus cells (CCs) accelerate oocyte aging by secreting soluble and heat-sensitive paracrine factors, the factors involved are not well characterized. Because Fas-mediated apoptosis represents a major pathway in induction of apoptosis in various cells, we proposed that CCs facilitate oocyte aging by releasing soluble Fas ligand (sFasL). In this study, we reported that when the aging of freshly ovulated mouse oocytes were studied *in vitro*, both the apoptotic rates of CCs and the amount of CCs produced sFasL increased significantly with the culture time. We found that oocytes expressed stable levels of Fas receptors up to 24 h of *in vitro* aging. Moreover, culture of cumulus-denuded oocytes in CCs-conditioned CZB medium (CM), in CZB supplemented with recombinant sFasL, or in CM containing sFasL neutralizing antibodies all showed that sFasL impaired the developmental potential of the oocytes whereas facilitating activation and fragmentation of aging oocytes. Furthermore, CCs from the *FasL*-defective gld mice did not accelerate oocyte aging due to the lack of functional FasL. In conclusion, we propose that CCs surrounding aging oocytes released sFasL in an apoptosis-related manner, and the released sFasL accelerated oocyte aging by binding to Fas receptors.

Mammalian oocytes are arrested at the meiotic metaphase II (MII) stage after ovulation. If not fertilized in time, the ovulated oocytes undergo a time-dependent process of aging^1^,^2^. *In vitro* culture of mature oocytes also leads to oocyte aging^3^,^4^,^5^,^6^. The postovulatory oocyte aging has marked detrimental effects on embryo development^5^,^7^,^8^,^9^ and offspring^10^,^11^. Aged oocytes also result in significant decrease in embryonic development following *in vitro* fertilization, intracytoplasmic sperm injection^12^ or nuclear transfer^13^,^14^,^15^. Thus, studies on mechanisms of oocyte aging are important for both normal and assisted reproduction.

Oocytes that mature both *in vivo* and *in vitro* are enclosed within cumulus cells (CCs), forming the so-called cumulus-oocyte-complexes (COCs). The CCs stay with *in vivo*-matured oocytes for different periods of times after ovulation, depending upon the species^1^,^3^,^16^,^17^, but the CCs will stay with *in vitro*-matured oocytes until artificially removed. The role of the surrounding CCs in oocyte maturation, ovulation and fertilization has been extensively studied^18^,^19^,^20^,^21^, yet work on their role in oocyte aging is limited. Although our previous studies indicated that CCs accelerate oocyte aging^6^,^22^,^23^, the underlying mechanisms are not fully understood. Notably, one of our studies showed that medium conditioned with CCs promoted aging of cumulus-denuded oocyte (DOs) *in vitro* but the aging-promoting effect is ablated when the conditioned medium (CM) was heated to 56°C for 15 min^22^. This suggests that CCs accelerate oocyte aging by secreting soluble and heat-sensitive factors. Furthermore, Wu et al.^24^ demonstrated that apoptotic CCs, in which extra-long BCL-2 interacting mediator of cell death (BIMEL) was up-regulated, accelerated porcine oocyte aging and degeneration *in vitro* via a paracrine manner. However, the oocyte aging-promoting factors involved in this process have yet to be characterized.

Fas ligand (FasL) is a type-II transmembrane protein that belongs to the tumor necrosis factor (TNF) family. Metalloproteinase mediated cleavage of transmembrane FasL results in the release of a soluble form (sFasL), which consists of the largest part of the extracellular domain of the FasL molecule^25^,^26^,^27^. Upon contact with FasL, cells expressing Fas undergo apoptosis rapidly by activating caspase-8 via Fas-Associated protein with a Death Domain (FADD)^28^. Fas-mediated apoptosis is a major pathway in the induction of apoptosis in various cells and tissues, which is important for both normal biological processes and pathological disorders^29^,^30^,^31^,^32^. In mice, expression of both *Fas* and *FasL* mRNA and their proteins were observed in granulosa cells of both normal and atretic follicles, but Fas was detected only in oocytes of atretic follicles^33^. In addition, Fas was expressed in immature bovine oocytes, whereas FasL was expressed in CCs^34^,^35^. Thus, reports on Fas expression in healthy oocytes remain to be verified. Furthermore, it is worthy of studying whether the Fas/FasL system plays any role in oocyte aging.

Mice homozygous for lpr (lymphoproliferation) or gld (generalized lymphoproliferative disease) develop lymphadenopathy and suffer from autoimmune disease. The lpr and gld are mutations in Fas and FasL, respectively^36^. The recombinant gld FasL expressed in COS cells could not induce apoptosis in cells expressing Fas. In reproduction, higher numbers of germ cells were found in fetal and postnatal ovaries of *Fas*-deficient mice than in age-matched wild-type mice^37^. As the mice aged, the ovarian size of lpr mice was much larger than that of wild-type mice due to increased numbers of ovarian follicles^38^. Further observations demonstrated that the apoptosis in wild-type mouse oocytes is achieved through the Fas receptor followed by the activation of caspase-3. In contrast, the aberrant expression and dysfunction of the mutant Fas receptor in lpr mouse oocytes were associated with an inability to activate caspase-3 and thus fail to induce nuclear DNA fragmentation^39^. However, effects of FasL (gld) mutations on oocytes have not been reported.

Based on the above-mentioned early studies, we proposed that accompanying oocye aging, CCs undergo apoptosis and the apoptotic CCs release sFasL that interact with Fas on oocytes to mediate oocyte aging, and that CCs from the gld mice with FasL mutations would not trigger oocyte aging due to their defective FasL. The objective of the present study was to test this hypothesis by using an *in vitro* aging system of oocytes as well as the oocytes from the gld mice with mutant FasL. As the apparent phenomenon of postovulatory-aged oocytes include impaired developmental potential^5^,^7^,^8^,^9^,^23^, increased susceptibility to activating stimuli^40^,^41^ and cytoplasmic fragmentation^42^, we used pre-implantation developmental potential and activation susceptibility as markers for early oocyte aging and cytoplasmic fragmentation as a marker for advanced oocyte aging.

## Results

### The Fas signaling pathway is active in aging oocytes

**Figure 1:**
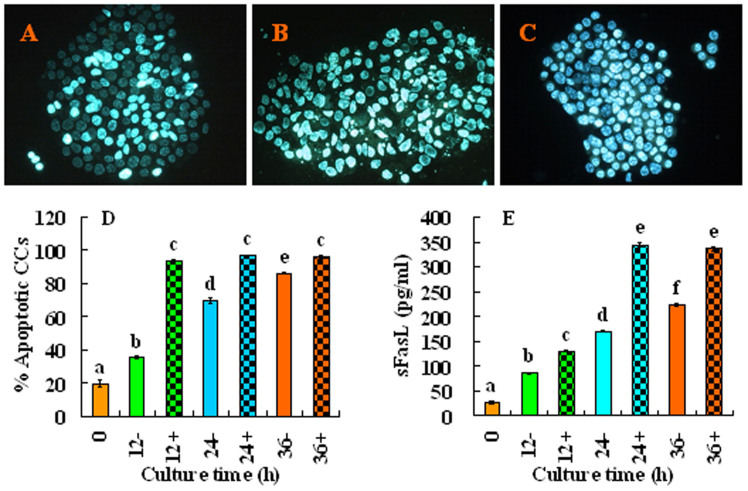
Effects of culture time and H_2_O_2_ on the apoptosis and sFasL release of CCs. CCs were cultured in regular CZB medium in the presence (+) or absence (−) of H_2_O_2_. At different times of the culture, the apoptotic rates in the CCs and the concentrations of sFasL in the CM were measured. Micrographs A, B and C show CCs smears stained with Hoechst 33342 and observed under a fluorescence microscope. The heterochromatin was heavily stained with Hoechst and gave bright fluorescence. Whereas the apoptotic cells showed pyknotic nuclei full of heterochromatin, healthy cells showed normal nuclei with sparse heterochromatin spots. Smears A, B and C show CCs collected at 0 h and 24 h of aging culture without or with H_2_O_2_, displaying approximately 20%, 75%, and 95% apoptotic cells, respectively. Original magnification ×400. Graphs D and E show the percentages of apoptotic CCs and levels of sFasL released by CCs after different treatments, respectively. Each treatment was repeated more than 3 times with each replicate containing 30 oocytes. a–e: Values without a common letter above their bars differ significantly (*P* < 0.05).

**Figure 2:**
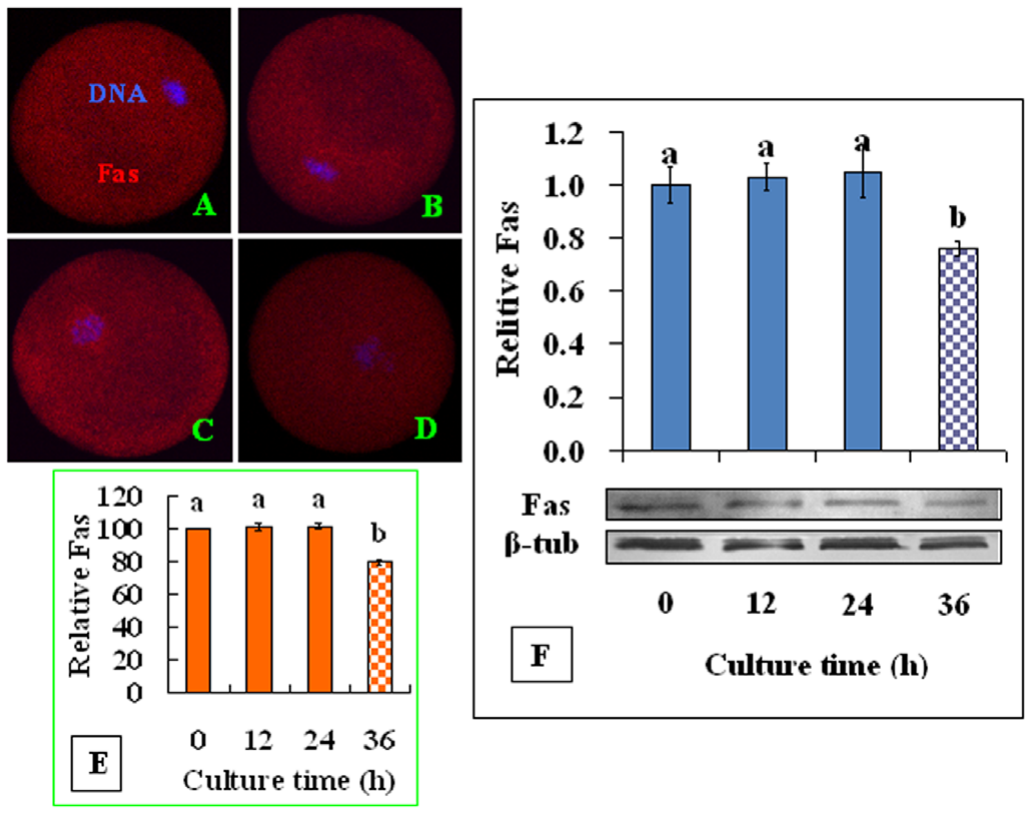
Levels of Fas receptor in aging oocytes. COCs were cultured in CZB medium for different times before examination for Fas localiztion and quantification by immunocytochemistry or Western blot analysis. A, B, C and D are confocal micrographs (original magnification ×400) showing Fas localization in oocytes that have aged for 0, 12, 24 and 36 h, respectively. E is a graph showing Fas quantification by immunocytochemistry in oocytes aged *in vitro* for different times. Each treatment was repeated 3–4 times with each replicate containing about 30 oocytes. F is a graph showing Fas levels by Western blot in oocytes aged for different times. β-tub: β-tubulin. a,b: Values with different letters above their bars differ significantly (*P* < 0.05).

To study whether the Fas pathway is active in aging oocytes, COCs or CCs were cultured in regular CZB medium in the presence or absence of H_2_O_2_. At different times of the culture, the apoptotic rates in CCs, the sFasL concentrations in CM conditioned with CCs, and Fas receptors levels in oocytes were measured. When CCs smears stained with Hoechst 33342 were observed under a fluorescence microscope, apoptotic cells show pyknotic nuclei that were full of heterochromatin, whereas healthy cells exhibit normal nuclei with sparse heterochromatin spots (Fig. 1A, B and C). Statistical analysis showed that both the apoptotic rates of CCs (Fig. 1D) and the sFasL contents (Fig. 1E) in CM conditioned with CCs increased significantly with culture time. At each time point of the culture, the presence of H_2_O_2_ further increased the apoptotic rates and sFasL secretion of the CCs. Immunohistochemical analysis revealed the expression of numerous Fas receptors on the aging oocytes (Fig. 2A-D). Quantification indicated that up to 24 h of culture the contents of Fas receptors in the oocytes remained constant, but the Fas receptor levels decreased significantly at 36 h of the culture (Fig. 2E). Western blot analysis revealed similar dynamics fluctuations of Fas receptors during oocyte aging (Fig. 2F). These results suggested that CCs released sFasL in an apoptotic state-related manner; thus, the maximal release was observed at 36 h of culture, and the presence of H_2_O_2_ further increased the apoptotic rates and the sFasL secretion of CCs. Oocytes possessed stable numbers of Fas receptors up to 24 h of *in vitro* aging.

### The sFasL released by apoptotic CCs impaired the developmental potential of aging oocytes

**Figure 3:**
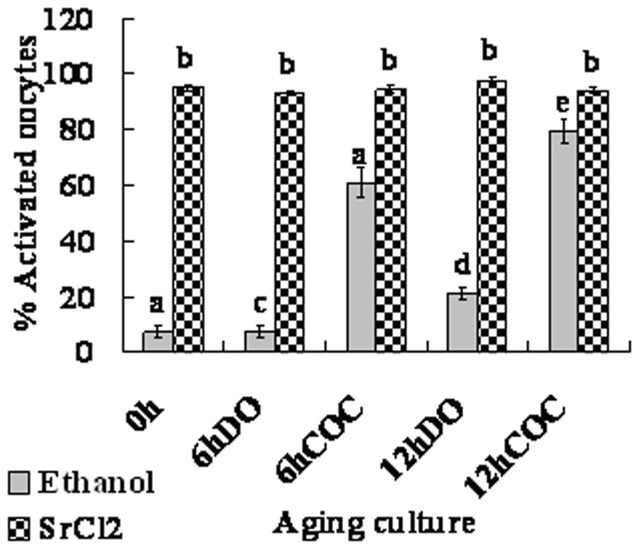
Oocyte activation following ethanol or SrCl_2_ treatment in DOs or COCs that had been aged in regular CZB medium for 0, 6 or 12 h. Each treatment was repeated 3–4 times with each replicate containing about 30 oocytes. a–e: Values without a common letter above their bars differ significantly (*P* < 0.05).

Routine work in our laboratory shows that while ethanol treatment is a weak stimulus that activates only the aged oocytes, Sr^2+^ treatment activates both aged and freshly-ovulated oocytes effectively. However, parthenotes developed better after Sr^2+^ treatment than after ethanol activation. An experiment was thus conducted to select proper methods for evaluation of oocyte activation susceptibility and for assessment of oocyte developmental potential, respectively. Freshly ovulated DOs or COCs were aged in regular CZB medium for different times before activation treatment with ethanol or SrCl_2_. Regardless of the ages of oocytes, 93% to 97% of the DOs or COCs were activated following Sr^2+^ treatment, but only 7% of freshly ovulated oocytes and about 20% of DOs or 80% of COCs aged *in vitro* for 12 h were activated following ethanol activation (Fig. 3). Thus, ethanol stimulus was used to evaluate the susceptibility of oocyte activation, and the Sr^2+^ stimulus was used to assess the development potential of oocytes in the present study.

**Figure 4:**
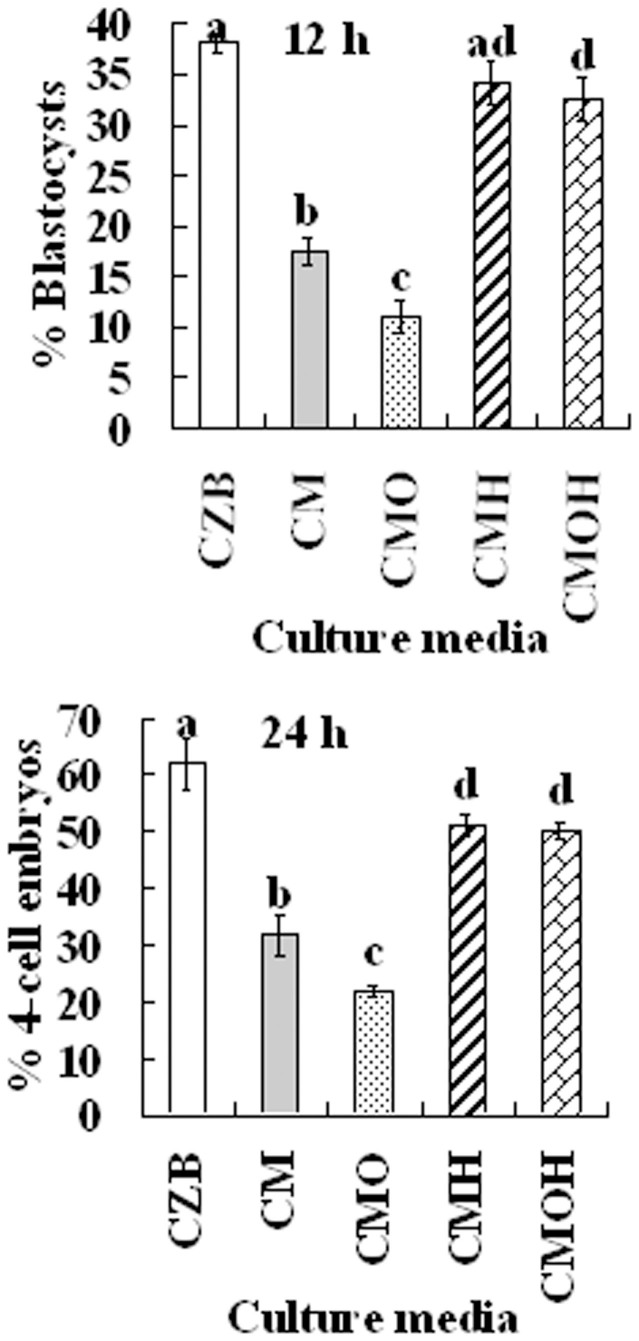
Development of Sr^2+^ activated embryos after mouse DOs collected 13 h post hCG were cultured for 12 h or 24 h in regular CZB or in different CM. To prepare CM, CZB was conditioned for 24 h with CCs that had been treated with (CMO) or without H_2_O_2_ (CM), and some of the CM collected was heated to 56°C to test the heat sensitivity of the aging-facilitating factor (CMH and CMOH). Each treatment was repeated more than 3 times with each replicate containing about 30 oocytes. a–f: Values without a common letter above their bars differ significantly (*P* < 0.05).

To test whether the Fas/FasL system plays a role in oocyte aging, we observed the impacts of different CM on the developmental potential of the oocytes. Freshly ovulated DOs were aged for 12 h or 24 h in different CM before Sr^2+^ -triggered activation for embryo development. To prepare CM of different types, CZB was conditioned for 24 h with CCs that had been treated with or without H_2_O_2_, and some of the produced CM was heated to 56°C to test the heat sensitivity of the sFasL. Our results showed that the percentages of oocytes that developed to 4-cell or blastocyst stage were significantly lower after culture in CM than that in CZB medium (Fig. 4). The CM produced from H_2_O_2_-treated CCs further reduced the developmental potential of aging oocytes. The heat treatment of CM significantly reduced its adverse effect on oocytes. Therefore, our results suggested that the sFasL released by apoptotic CCs induced oocyte aging and was heat-sensitive.

### Supplement of aging-culture medium with sFasL impaired the developmental potential of aging oocytes

**Figure 5:**
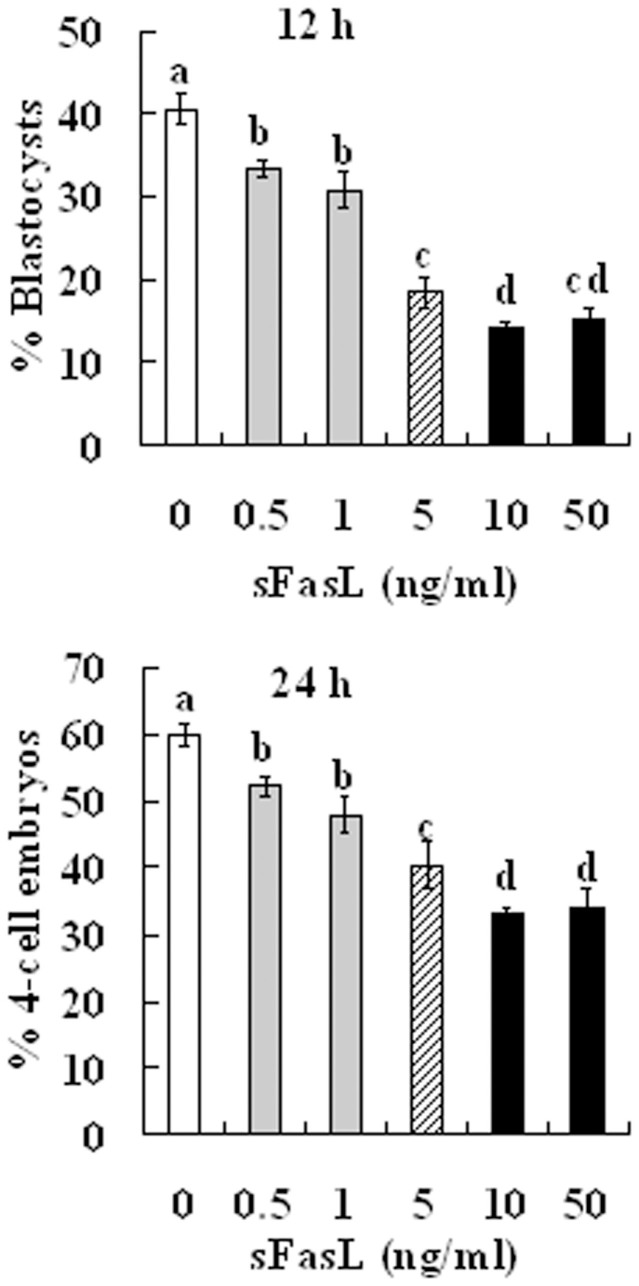
Development of Sr^2+^-activated embryos after mouse DOs collected 13 h post hCG were cultured for 12 h or 24 h in CZB medium containing different concentrations of sFasL. Each treatment was repeated more than 3 times with each replicate containing 30 oocytes. a–d: Values without a common letter above their bars differ significantly (*P* < 0.05).

To further confirm that the apoptotic CCs promoted oocyte aging by releasing sFasL, freshly ovulated DOs were cultured for 12 or 24 h in CZB medium that was supplemented with different concentrations of recombinant sFasL before activation with Sr^2+^ for embryo development. When cultured for 12 h, the blastocyst rates decreased significantly with increasing concentrations of sFasL (Fig. 5). However, the lowest blastocysts rate (about 15%) was not achieved until the supplemented sFasL was increased to 10 ng/ml, a concentration that was 30–50 times higher than that (0.2–0.3 ng/ml) contained in the CM that achieved the same low level of blastocyst rates following conditioning with H_2_O_2_-treated CCs for 24 h (Fig. 4). When cultured for 24 h, although no blastocysts were observed, the percentages of activated oocytes that developed into 4-cell embryos decreased significantly with increasing sFasL concentrations, which reached the lowest level by 10 ng/ml of sFasL (Fig. 5).

### Effects of neutralizing antibodies against sFasL on the capacity of CM to induce oocyte aging

**Figure 6:**
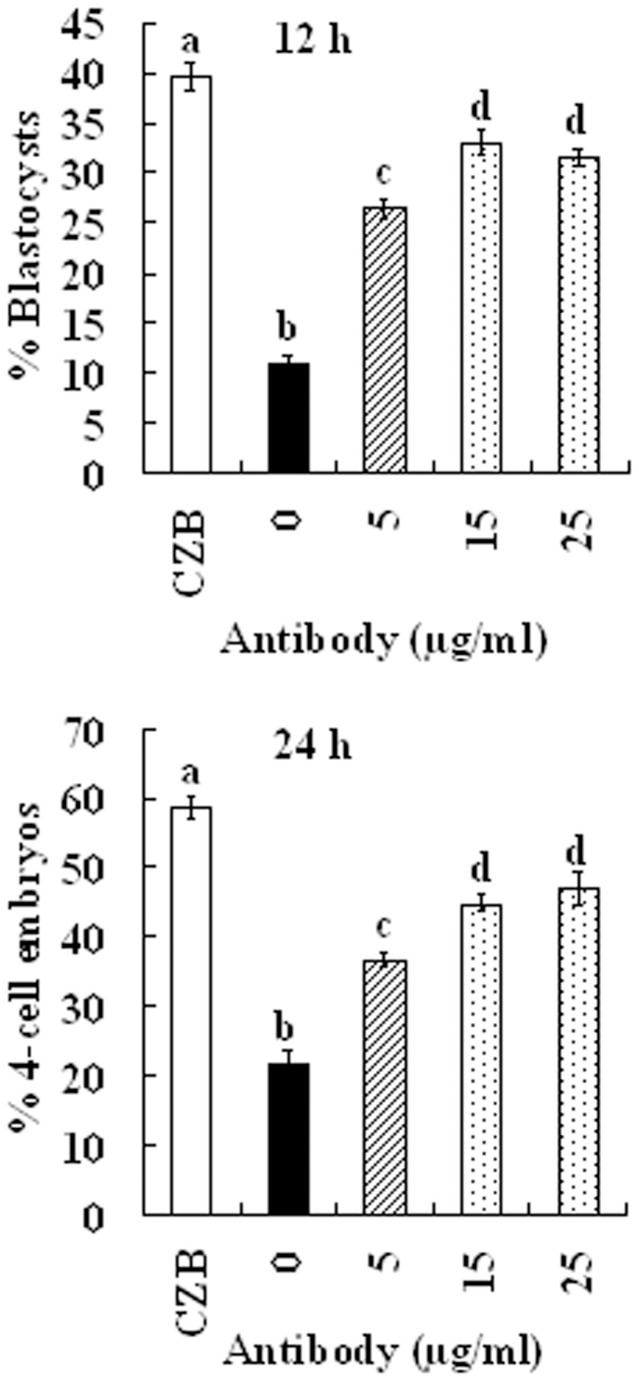
Effects of FasL antibodies containing CM on the development of Sr^2+^-activated oocytes. CM conditioned for 24 h with H_2_O_2_-treated CCs was neutralized for 6 h at 37°C with different concentrations of FasL antibodies. DOs collected 13 h post hCG were aged for 12 h or 24 h in the neutralized CM before Sr^2+^ activation was applied. Each treatment was repeated 3–4 times with each replicate containing about 30 oocytes. a–d: Values without a common letter above their bars differ significantly (*P* < 0.05).

To obtain further evidence that sFasL was the major factor in the CM that induced oocyte aging, CM conditioned for 24 h with H_2_O_2_-treated CCs was neutralized for 6 h at 37°C with different concentrations of FasL antibodies. Freshly ovulated DOs collected 13 h after hCG were aged for 12 or 24 h in the neutralized CM before Sr^2+^ activation for embryo development. Our results showed that the rates of blastocysts and 4-cell embryos increased with increasing concentrations of FasL antibodies (Fig. 6), but the highest rates of blastocysts (32%) and 4-cell embryos (46%) obtained at 15 μg/ml were still significantly lower than the oocytes aged in CZB alone (39% and 59%, respectively). These results suggested that sFasL was the major factor in the CM that induced oocyte aging, and in addition to sFasL, the CM may also contain other factors that induced oocyte aging.

### Effects of sFasL on the activation of aging oocytes

To determine the effects of sFasL in oocyte aging, the susceptibility of oocytes to activation reagents was observed. Newly ovulated DOs were aged for 12 h in CZB alone, CZB containing 10 ng/ml sFasL, CM conditioned with CCs, or CM conditioned with H_2_O_2_-treated CCs before ethanol treatment was applied for oocyte activation. The results showed that compared to only 21.5 ± 2.0% (n = 121) of the oocytes aged in CZB were activated, the activation rates increased significantly (*P* < 0.05) to 38.3 ± 2.7% (n = 133), 34.3 ± 2.0 (n = 120), and 57.0 ± 1.2% (n = 120) in oocytes aged in CZB containing 10 ng/ml sFasL, in CCs-conditioned CM and in CM conditioned with H_2_O_2_-treated CCs, respectively. These results suggested that sFasL induced oocyte aging and CM, particularly CM conditioned with H_2_O_2_-treated CCs, was more effective than supplement with 10 ng/ml sFasL in inducing oocyte aging.

### Effects of sFasL on cytoplasmic fragmentation of aging oocytes

**Figure 7:**
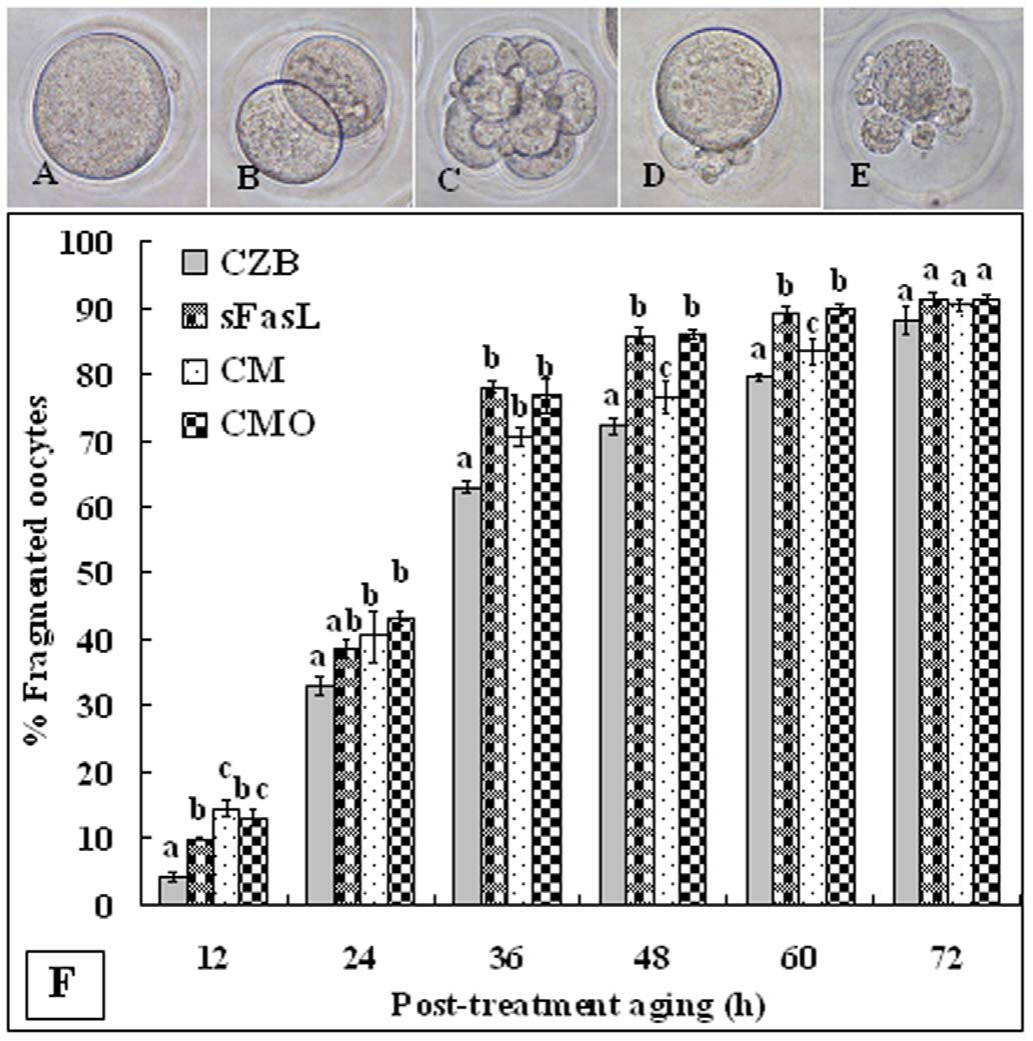
Fragmentation of aging oocytes. DOs collected 13 h post hCG were cultured for 24 h in CZB alone, CZB with 10 ng/ml sFasL, CM conditioned for 24 h with CCs (CM) or H_2_O_2_-treated CCs (CMO) before post-treatment aging in CZB alone. At different times of the post-treatment aging, oocytes were observed for fragmentation. Photographs A–E show aged oocytes with different fragmenting patterns. F is graph showing percentages of fragmented oocytes at different times of post-treatment aging. Each treatment was repeated 3–4 times with each replicate containing about 30 oocytes. a–c: Values without a common letter above their bars differ significantly (P < 0.05) within time points of post-treatment aging.

To study the effects of sFasL in cytoplasmic fragmentation of aging oocytes, newly ovulated DOs were treated for 24 h in CZB alone, CZB containing 10 ng/ml sFasL, CM conditioned with CCs (CM), or CM conditioned with H_2_O_2_-treated CCs (CMO) before post-treatment aging in CZB alone. At different times of the post-treatment aging, oocytes were observed for fragmentation. Oocytes with a clear moderately granulate cytoplasm and an intact first polar body were considered to be un-fragmented (Fig. 7A), and oocytes with more than two asymmetric cells were considered to be fragmented (Fig. 7B-E). Statistical analysis showed that control oocytes that were pre-cultured in CZB alone began fragmentation at 12 h of post-treatment aging, and the fragmentation rates increased significantly thereafter (Fig. 7F). Oocytes pre-cultured in CZB containing sFasL or in CM or CMO showed significantly higher fragmentation rates than the control oocytes at different times of post-treatment aging (Fig. 7F). These results suggested that sFasL triggered cytoplasmic fragmentation of aging oocytes, and CM conditioned with H_2_O_2_-treated CCs exhibited a higher capacity of inducing oocyte fragmentation than supplement with 10 ng/ml sFasL.

### Experiments using the gld mice lacking a functional FasL

**Figure 8:**
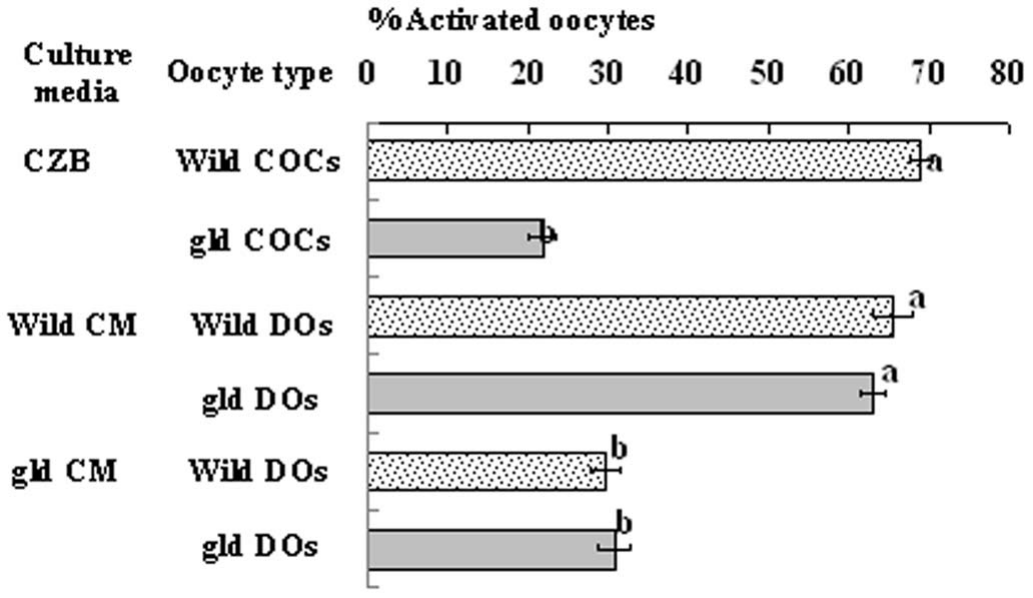
Ethanol-activation rates of oocytes collected from wild-type or gld mice. The oocytes were collected at 13 h post hCG injection and were cultured for 6 h as COCs in CZB medium, or for 12 h as DOs in CM prepared with wild-type or gld CCs. Each treatment was repeated 3 times with each replicate containing about 25 oocytes. a–b: Values with different letters in their bars differ significantly (*P* < 0.05).

To further study the roles of the Fas system in mediating oocyte aging, we introduced the *FasL*-defective gld mice and performed two experiments. In the first experiment, COCs from wild-type or gld mice were aged for 6 h in regular CZB medium before ethanol treatment was applied for observation of activation. We found that about 70% of the COCs from wild-type mice were activated, but only 20% of the oocytes from gld mice were activated following ethanol treatment (Fig. 8). In the second experiment, DOs from wild-type or gld mice were aged for 12 h in CM conditioned with H_2_O_2_-treated CCs from either wild-type or gld mice before ethanol treatment. When aged in CM conditioned with wild-type CCs, oocytes from both wild-type and gld mice showed activation rates of over 60% after the ethanol treatment (Fig. 8). When aged in CM conditioned with gld CCs, however, both wild-type and gld oocytes showed low activation rates of about 30% following ethanol stimulation. These results suggested that CCs from the gld mice did not induce oocyte aging due to their defective FasL, this further confirms the role of the Fas/FasL pathway in facilitating oocyte aging.

## Discussion

The present study suggested that the Fas/FasL pathway was established in freshly ovulated MII mouse oocytes, while the oocyte expressed maximum numbers of Fas receptors, the CCs released sFasL. Dharma et al.^33^ observed expression of both Fas and FasL mRNA and protein in granulosa cells of healthy and atretic follicles. However, although oocytes in atretic follicles showed intense expression of Fas, oocytes in healthy follicles did not show any Fas expression. Similarly, Guo et al.^43^,^44^ reported that the expression of both Fas and FasL in mouse ovarian follicle was restricted in granulosa cells and no expression was observed in oocytes. The different reports on Fas expression in healthy oocytes may be caused by the different maturation stages of the oocytes or gonadotropin stimulation. For example, Mori et al.^45^ observed positive expression of Fas in both intra-ovarian oocytes and hyper-ovulated eggs following eCG stimulation. According to Guo et al.^43^,^44^, the highest level of *FasL* mRNA was observed in murine ovaries and granulosa cells 1 day after the administration of eCG, while the level of *FasL* mRNA became very weak at day 5. Furthermore, using the same immunohistochemical procedures for ovulated oocytes, we found that immature oocytes isolated from ovarian follicles of adult mice after eCG stimulation showed intense Fas expression, whereas Fas expression was almost undetectable in oocytes isolated from unstimulated prepubertal (17 days old) mice (data not shown).

In this study, when freshly ovulated oocytes were aged *in vitro* for different times, both the apoptotic rates of CCs and the amount of sFasL released by CCs increased significantly with the culture time, and the presence of H_2_O_2_ in culture medium further increased the apoptosis and sFasL production of CCs. This suggests that higher levels of sFasL were released by the CCs with their increasing degrees of apoptosis. Earlier studies have reported that co-incubation with human neutrophils that underwent spontaneous apoptosis significantly induced the apoptosis in A549 pulmonary adenocarcinoma cells via releasing sFasL^46^. The cisplatin-stimulated plaa (high) cells, which contained significantly higher levels of DNA fragmentation, caspase activities, PLA(2) enzyme activity, and cytochrome C leakage from mitochondria, displayed higher levels of phosphorylated JNK/c-Jun and FasL, as compared to plaa (low) cells that were treated the same way^47^. Also, oxidative stress induces the expression of Fas and FasL and apoptosis in murine intestinal epithelial cells^48^. Furthermore, oxidative stress and hypoxia/reoxygenation trigger CD95 (APO-1/Fas) ligand expression in microglial cells^49^.

The present results showed that the level of Fas receptors on oocytes remained stable up to 24 h of *in vitro* aging. This suggested that up to 24 h of culture, the aging oocytes had sufficient amount of Fas receptors to interact with the increasing levels of sFasL to induce oocyte aging. The incubation of DOs in CCs-conditioned CZB or in CZB supplemented with recombinant sFasL showed that sFasL impaired the developmental competence of the oocytes but mediate the activation and fragmentation of the aging oocytes. Furthermore, by using the gld mice that carry mutations of FasL, the present work showed that the CCs from these mice did not trigger oocyte aging due to their defective FasL, confirming further the important role of the Fas system in facilitating oocyte aging. Co-culture with polymorphonuclear neutrophils in a trans-well system induced apoptosis in human alveolar epithelial (A549) cells^50^, a process that was also mediated by sFasL^46^. Previous studies showed that oocyte aging led to apoptotic cell death. For example, the expression of anti-apoptotic protein BCL2 was gradually reduced during oocyte aging^23^,^51^,^52^. Fertilization-like Ca^2+^ responses induced by injection of sperm cytosolic factor triggered cell death, rather than activation, in aged oocytes. These oocytes exhibited not only extensive cytoplasmic and DNA fragmentation, but also a prominent decreased Bcl-2 and activated caspases^42^,^51^,^53^. Taken together, the current study has provided evidence that CCs of aging oocytes release sFasL that accelerates oocyte aging through binding to Fas receptors.

In the present study, the CM conditioned for 24 h with H_2_O_2_-treated CCs containing about 30–50 folds less sFasL showed the same or even higher capacity of inducing oocyte aging than supplementation with 10 ng/ml sFasL. Naturally processed and recombinant sFasL were found to induce apoptosis in mouse somatic cells at a concentration of 10 ng/ml^25^,^26^,^54^,^55^. Why is such a large amount of the recombinant sFasL required to induce apoptosis? It is known that an uncharacterized metalloproteinase cleaves the 40-kD membrane-bound FasL to generate the 26–29 kD-soluble fragment^26^,^54^ and that aggregated or membrane-bound FasL was much more active than the trimeric sFasL. For example, the apoptosis-inducing activity of processed sFasL after its purification and removal of residual membrane-bound FasL by Triton X-114 extraction was marginal^56^. Recombinant sFasL that is of similar length to the naturally processed form was equally inactive.

Then, why supernatants of cells expressing FasL were so pro-apoptotic? One well-described example is the highly apoptotic supernatant of Neuro-2a cells transfected with murine FasL^57^, which induces apoptosis in human Jurkat or mouse A20 cells at concentrations as low as 1 ng/ml. Western blot analysis revealed that the FasL detected in these supernatants was not the processed sFasL, but was the membrane-bound, unprocessed form^56^. According to Schneider et al.^56^, it is likely that Neuro-2a cells express little or no processing proteases, and that membrane fragments or vesicles containing the membrane-bound FasL are released into the supernatant. Thus, the present results suggest that FasL released by apoptotic CCs are mainly membrane-bound FasL released as membrane fragments or vesicles. Another possibility for the high efficiency of CM to induce oocyte aging is that in addition to sFasL, the apoptotic CCs might secrete other proteins that mediate oocyte aging as well. Our FasL antibody neutralization test, which showed that the highest blastocysts rates obtained following maximum neutralization of CM with FasL antibodies were still significantly lower than oocytes aged in CZB alone, confirmed this expectation. The soluble tumor necrosis factor (TNF)-related apoptosis-inducing ligand (sTRAIL), another member of the TNF ligand family, has been reported in granulosa cells of porcine^58^ and human ovaries^59^, and high level of TRAIL was expressed in CCs from diabetic mice that showed more apoptotic follicles compared to controls, suggesting that this apoptotic pathway is up-regulated in oocytes under hyperglycemic stress^60^.

In summary, we propose that CCs surrounding aging oocytes released sFasL in an apoptosis-related manner, and the released sFasL accelerated oocyte aging by binding to Fas receptors. We believe that our data are important for understanding the mechanisms not only for oocyte aging but also for the Fas/FasL signaling. Further studies on how sFasL promotes oocyte activation would provide novel insights into the mechanisms by which Fas signaling regulates cell cycle and apoptosis. In addition, the present results indicated that oocyte aging without H_2_O_2_ treatment also led to CCs apoptosis and production of sFasL, which induced oocyte aging through binding to Fas receptors. Thus, the knowledge from this study may also have clinical implications for assisted-reproduction techniques because it may improve the *in vitro* treatment of mature oocytes. For example, protocols maybe developed or improved to prevent oocyte aging *in vitro* through inhibiting CCs apoptosis or by preserving DOs before fertilization or other manipulations *in vitro*.

## Methods

Chemicals and reagents used in the present study were purchased from Sigma Chemical Co. unless otherwise specified.

### Oocyte recovery

Mice for most of the experiments in this study are the Kunming breed, which were bred in our laboratory. The gld mice with a germline mutation F273L in FasL in a C57BL/6J genomic background were obtained from the Key Laboratory of Stem Cell Biology, Shanghai Institute for Biological Sciences, China. Wild-type C57BL/6J mice were purchased from Shandong University Center for Laboratory Animals. The mice were kept in a room under a 14L:10D photoperiod, with lights-off at 20:00 h. All procedures for mouse care and use were conducted in accordance with the guidelines and approved by the Animal Research Committee of the Shandong Agricultural University, P. R. China (Permit number: 20010510).

Young adult female mice (6–8 weeks of age) were superovulated with 10 IU equine chorionic gonadotropin (eCG, i.p.) that was followed by 10 IU human chorionic gonadotropin (hCG, i.p.) 48 h later. Both eCG and hCG were purchased from Ningbo Hormone Product Co., Ltd., China. The superovulated mice were sacrificed 13 h after hCG injection, and the COCs were recovered from the oviducts. After dispersing and washing three times in M2 medium, some of the COCs were denuded of cumulus cells by pipetting with a thin pipette in a drop of M2 medium containing 0.1% hyaluronidase to prepare cumulus-denuded oocytes (DOs).

### Preparation of CCs and conditioned media (CM)

To prepare regular CCs, the CCs isolated during the preparation of DOs were washed twice by centrifugation (200 × g, 5 min each) in regular CZB medium (NaCl, 81.62 mM; KCl, 4.83 mM; KH_2_PO_4_, 1.18 mM; MgSO_4_, 1.18 mM; NaHCO_3_, 25.12 mM; CaCl_2_·2H_2_O, 1.7 mM; sodium lactate, 31.3 mM; sodium pyruvate, 0.27 mM; EDTA, 0.11 mM; glutamine, 1 mM; bovine serum albumin, 5 g/L; penicillin, 0.06 g/L; streptomycin, 0.05 g/L). The pellets were then resuspended in a proper volume of CZB to obtain a final CCs suspension of 5–8 × 10^5^ cells/ml. To prepare H_2_O_2_-treated CCs, the CCs pellets were resuspended in CZB medium containing 200 μM H_2_O_2_, and the cell suspension was added to a 96-well culture plate (200 μl per well) and incubated at 37.5°C for 24 h in a humidified atmosphere containing 5% CO_2_. At the end of the treatment, CCs were harvested and washed by centrifugation and the pellets were resuspended with regular CZB before being used for medium conditioning.

To prepare CM, regular or H_2_O_2_-treated CCs were cultured in regular CZB medium (200 μl per well) for 24 h. At the end of culture, the culture medium was aspirated from the wells and centrifuged at 300 × g for 5 min to remove cells and debris. The CM obtained was frozen at −80°C until use. Before use, some of the CM was heated to 56°C for 10 min to denature the oocyte aging-promoting factors. To neutralize the sFasL in CM, anti-FasL antibodies (Abcam) were added to CM at different concentrations and incubated at 37.5°C for 6 h.

### *In vitro* aging of oocytes

For *in vitro* aging, COCs or DOs were cultured in regular CZB medium or CM for different times. Briefly, CZB or CM was placed in wells of a 96-well culture plate (100 μl per well). About 30 COCs or DOs were transferred to each well, covered with mineral oil, and cultured for different times at 37.5°C under 5% CO_2_ in humidified air. To study the effect of recombinant sFasL, a stock solution of recombinant mouse sFasL (100 μg/ml) was prepared by dissolving the recombinant sFasL (R&D System) in sterile PBS containing 0.1% bovine serum albumin. The stock solution prepared was stored at −20°C until use. The recombinant mouse sFasL was added to CZB medium at different concentrations.

### Assessment of CCs apoptosis

The CCs obtained from COCs or recovered from cell culture were separated by centrifugation (200 × g, 5 min at room temperature). The CCs pellets were resuspended in 50 μl of M2 medium supplemented with 0.01 mg/ml of Hoechst 33342 and stained in the dark for 5 min. The stained cells were then centrifuged for 5 min at 200 × g. After removal of approximately half the supernatant, a 5-μl drop of suspension was smeared on a slide and observed under a Leica DMLB fluorescence microscope (400×). Six to eight fields were randomly examined on each smear, and the percentages of apoptotic cells were calculated from 60–80 cells observed in each field. All images were reviewed by 2 investigators in a double blind manner.

### Enzyme-linked immunosorbent assay (ELISA) for sFasL

To measure sFasL contents in the culture medium, ELISA was conducted using a Mouse Factor Related Apoptosis Ligand (FASL) Elisa kit (BLUE GENE, Shanghai, China). Briefly, 50 μl of standards or samples were added in duplicate to wells of a micro-titer plate pre-coated with mouse monoclonal antibodies, then 50 μl of Conjugate was added to each well and incubated for 60 min at 37°C. After the micro-titer plate was washed using the wash solution and dried using paper towels, 50 μl of Substrates A and 50 μl of Substrates B were added to each well and incubated for 15 min at 25°C. The optical density was measured at 450 nm using a plate reader (BioTek-ELx808, BioTek Instruments, Inc.) within 15 min after the reaction was terminated by 50 μl of Stop Solution. The concentrations of FasL in the culture medium were calculated according to the standard curves.

### Immunofluorescence microscopy

All the procedures were performed at room temperature unless otherwise specified. Cumulus-free oocytes were washed 3 times in M2 medium between treatments. Oocytes were (i) fixed with 3.7% paraformaldehyde in PHEM buffer (60 mM Pipes, 25 mM Hepes, 10 mM EGTA and 4 mM MgSO_4_, pH 7.0) for at least 30 min, followed by treatment with 0.25% protease in M2 for 1 to 2 seconds to remove zona pellucida; (ii) permeabilized with 0.1% Triton X-100 in PHEM for 5 min; (iii) blocked in PHEM containing 3% BSA for 1 h; (iv) incubated at 4°C overnight with mouse monoclonal anti-Fas (IgG, 1:200, Abcam) in 3% BSA in M2 medium; (v) incubated for 1 h with Cy3-conjugated goat-anti-rabbit IgG (1:800, Jackson ImmunoResearch) in 3% BSA in M2; (vi) incubated for 10 min with 10 μg/ml Hoechst 33342 in M2. Negative control samples in which the primary antibody was omitted were also processed. The stained oocytes were mounted on glass slides and observed with a Leica laser scanning confocal microscope (TCS SP2). Helium/neon (He/Ne; 543 nm) lasers were used to excite Cy3, fluorescence was detected with bandpass emission filter (560–605 nm), and the captured signals were recorded as red. The relative content of Fas was quantified by measuring the fluorescence intensities. For each experimental series, all high-resolution z-stack images were acquired with identical settings. The relative intensities were measured on the raw images using Image-Pro Plus software (Media Cybernetics Inc., Silver Spring, MD) under fixed thresholds across all slides.

### Western blot analysis

Two hundred DOs were placed in a 1.5 ml microfuge tube containing 20 μl sample buffer (20 mM Hepes, 100 mM KCl, 5 mM MgCl_2_, 2 mM DTT, 0.3 mM PMSF, 3 mg/ml leupetin, pH 7.5) and frozen at −80°C until use. For running the gel, 5 μl of 5× SDS-PAGE loading buffer was added to each tube and the tubes were heated at 100°C for 5 min. The samples were separated on a 12% SDS-PAGE and transferred onto PVDF membranes. The membranes were washed twice in TBST (150 mM NaCl, 2 mM KCl, 25 mM Tris, 0.05% Tween-20, pH 7.4) and blocked with TBST containing 3% BSA at room temperature for 1–1.5 h. The membranes were then incubated at 4°C overnight with primary antibodies at a dilution of 1:1000 in 3% BSA-TBST. After being washed three times in TBST (5 min each), the membranes were incubated for 1 h at 37°C with second antibodies diluted to 1:1000 in 3% BSA-TBST. After three washings in TBST, the membranes were detected by a BCIP/NBT alkaline phosphatase color development kit (Beyotime Institute of Biotechnology, China). The relative quantities of proteins were determined with Image J software by analyzing the sum density of each protein band image. The values of freshly-ovulated oocytes were set as 100% and the other values were expressed relative to this quantity. β-tubulin was used as internal controls. The primary antibodies used included rabbit polyclonal to Fas (Abcam, ab82419) and mouse anti-β-tubulin (Merck Millipore, 05-661). The secondary antibodies included goat anti-rabbit IgG AP conjugated (CWBIO, cw0111) and goat anti-mouse IgG AP conjugated (CWBIO, cw0110).

### Activation of oocytes

In this study, ethanol stimulus was used to evaluate oocyte activation susceptibility, and the Sr^2+^ stimulus was used to assess the developmental potential of oocytes. For ethanol activation, oocytes were first treated with 5% (v/v) ethanol in M2 medium for 5 min at room temperature, then washed three times, and cultured in regular CZB medium containing 2 mM 6-dimethylaminopurine for 6 h at 37.5°C in a humidified atmosphere containing 5% CO_2_ in air. The activating medium used for Sr^2+^ activation was Ca^2+^-free CZB supplemented with 10 mM SrCl_2_ and 5 μg/ml cytochalasin B. After washing twice in M2 medium and once in the activating medium, the oocytes were incubated in the activating medium for 6 h. At the end of the activation culture, oocytes were examined under a microscope for activation. Only those oocytes that had one or two pronuclei, or two cells each having a nucleus, were considered activated.

### Embryo culture

Activated oocytes were cultured for 4 days in regular CZB medium (about 30 oocytes in a 100 μl drop) at 37.5°C under humidified atmosphere containing 5% CO_2_ in air. Glucose (5.55 mM) was added to CZB medium when the embryos were cultured beyond 3- or 4-cell stages.

### Assessment of oocyte fragmentation

At different times of the culture, DOs were examined under a phase contrast microscope for morphological feature. Oocytes with a clear moderately granulate cytoplasm and an intact first polar body were considered un-fragmented. Oocytes with more than two asymmetric cells were considered fragmented.

### Data analysis

At least three replicates were used for each treatment. Percentage data were arcsine transformed and analyzed with ANOVA; a Duncan multiple comparison test was used to locate differences. The software used was the Statistics Package for Social Science (SPSS, Inc.). Data were expressed as the mean ± SEM, and *P* < 0.05 was considered to be significant.

## Author Contributions

J.Zhu, J.Zha., H.L., T.Y.W., C.X.Z. and M.J.L. performed the experiments; J.Zhu and J.H.T. analyzed the data; J.H.T. designed the experiments and wrote the manuscript. All authors reviewed the manuscript.

## References

[b1] Yanagimachi R. & Chang M. C. Fertilizable life of golden hamster ova and their morphological changes at the time of losing fertilizability. J Exp Zool 148, 185–203 (1961). 14008938 10.1002/jez.1401480303

[b2] Whittingham D. G. & Siracusa G. The involvement of calcium in the activation of mammalian oocytes. Exp Cell Res 113, 311–317 (1978). 299649 10.1016/0014-4827(78)90371-3

[b3] Longo F. J. Aging of mouse eggs *in vivo* and *in vitro*. Gamete Res 3, 379–393 (1980).

[b4] Webb M., Howlett S. K. & Maro B. Parthenogenesis and cytoskeletal organization in aging mouse eggs. J Embryol Exp Morphol 95, 131–145 (1986). 3794588

[b5] Tarin J. J., Ten J., Vendrell F. J. & Cano A. Dithiothreitol prevents age associated decrease in oocyte/conceptus viability *in vitro*. Hum Reprod 13, 381–386 (1998). 9557843 10.1093/humrep/13.2.381

[b6] Miao Y. L. *et al.* Cumulus cells accelerate aging of mouse oocytes. Biol Reprod 73, 1025–1031 (2005). 15987819 10.1095/biolreprod.105.043703

[b7] Juetten J. & Bavister B. D. Effects of egg aging on *in vitro* fertilization and first cleavage division in the hamster. Gamete Res 8, 219–230 (1983).

[b8] Tesarik J. Subzonal sperm insertion with aged human oocytes from failed *in vitro* fertilization attempts: fertilization results and some applications. Hum Reprod 8, 1245–1250 (1993). 8408522 10.1093/oxfordjournals.humrep.a138235

[b9] Winston N. J., Braude P. R. & Johnson M. H. Are failed-fertilized human oocytes useful? Hum Reprod 8, 503–507 (1993). 8501174 10.1093/oxfordjournals.humrep.a138084

[b10] Tarin J. J. *et al.* Long-term effects of postovulatory aging of mouse oocytes on offspring: a two-generational study. Biol Reprod 61, 1347–1355 (1999). 10529284 10.1095/biolreprod61.5.1347

[b11] Tarin J. J., Perez-Albala S., Perez-Hoyos S. & Cano A. Postovulatory aging of oocytes decreases reproductive fitness and longevity of offspring. Biol Reprod 66, 495–499 (2002). 11804967 10.1095/biolreprod66.2.495

[b12] Lacham-Kaplan O. & Trounson A. Reduced developmental competence of immature, in-vitro matured and postovulatory aged mouse oocytes following IVF and ICSI. Reprod Biol Endocrinol 6, 58 (2008). 19040764 10.1186/1477-7827-6-58 PMC2636812

[b13] Cervera R. P. & Garcı'a-Xime'nez F. Oocyte age and nuclear donor cell type affect the technical efficiency of somatic cloning in rabbits. Zygote 11, 151–158 (2003). 12828414 10.1017/s0967199403002181

[b14] Iwamoto M. *et al.* Effects of caffeine treatment on aged porcine oocytes: parthenogenetic activation ability, chromosome condensation and development to the blastocyst stage after somatic cell nuclear transfer. Zygote 13, 335–345 (2005). 16388702 10.1017/S0967199405003370

[b15] Wu Y. G. *et al.* The effects of delayed activation and MG132 treatment on nuclear remodeling and preimplantation development of embryos cloned by electrofusion are correlated with the age of recipient cytoplasts. Cloning Stem Cells 9, 417–431 (2007). 17907952 10.1089/clo.2006.0023

[b16] Longo F. J. An ultrastructural analysis of spontaneous activation of hamster eggs aged *in vivo*. Anat Rec 179, 27–55 (1974). 4362489 10.1002/ar.1091790104

[b17] Tan J. H. An ultrastructural study on the pig oocyte during its aging after ovulation. Acta Vet Zootech Sinica 16, 1–4 (1985).

[b18] Eppig J. J. The relationship between cumulus cell-oocyte coupling, oocyte meiotic maturation, and cumulus expansion. Dev Biol 89, 268–272 (1982). 7054011 10.1016/0012-1606(82)90314-1

[b19] Eppig J. J. Intercommunication between mammalian oocytes and companion somatic cells. Bioessays 13, 569–574 (1991). 1772412 10.1002/bies.950131105

[b20] Buccione R., Schroeder A. C. & Eppig J. J. Interactions between somatic cells and germ cells throughout mammalian oogenesis. Biol Reprod 43, 543–547 (1990). 2289008 10.1095/biolreprod43.4.543

[b21] Tanghe S., Van Soom A., Nauwynck H., Coryn M. & de Kruif A. Minireview: functions of the cumulus oophorus during oocyte maturation, ovulation, and fertilization. Mol Reprod Dev 61, 414–424 (2002). 11835587 10.1002/mrd.10102

[b22] Qiao T. W. *et al.* Cumulus cells accelerate aging of mouse oocytes by secreting a soluble factor(s). Mol Reprod Devel 75, 521–528 (2008). 17886273 10.1002/mrd.20779

[b23] Liu N. *et al.* Pyruvate prevents aging of mouse oocytes. Reproduction 138, 223–234 (2009). 19465488 10.1530/REP-09-0122

[b24] Wu Y. *et al.* BIM EL-mediated apoptosis in cumulus cells contributes to degenerative changes in aged porcine oocytes via a paracrine action. Theriogenology 76, 1487–1495 (2011). 21835451 10.1016/j.theriogenology.2011.06.016

[b25] Kayagaki N. *et al.* Metalloproteinase-mediated release of human Fas ligand. J Exp Med 182, 1777–1783 (1995). 7500022 10.1084/jem.182.6.1777 PMC2192231

[b26] Tanaka M., Suda T., Takahashi T. & Nagata S. Expression of the functional soluble form of human fas ligand in activated lymphocytes. EMBO J 14, 1129–1135 (1995). 7536672 10.1002/j.1460-2075.1995.tb07096.x PMC398190

[b27] Mitsiades N., Poulaki V., Kotoula V., Leone A. & Tsokos M. Fas ligand is present in tumors of the Ewing's sarcoma family and is cleaved into a soluble form by a metalloproteinase. Am J Pathol 153, 1947–1956 (1998). 9846984 10.1016/S0002-9440(10)65708-2 PMC1866328

[b28] Itoh N. *et al.* The polypeptide encoded by the cDNA for human cell surface antigen Fas can mediate apoptosis. Cell 66, 233–243 (1991). 1713127 10.1016/0092-8674(91)90614-5

[b29] Dhein J., Walczak H., Baumler C., Debatin K. M. & Krammer P. H. Autocrine T-cell suicide mediated by APO-1/(Fas/CD95). Nature 373, 438–441 (1995). 7530335 10.1038/373438a0

[b30] Ju S. T. *et al.* Fas(CD95)/FasL interactions required for programmed cell death after T-cell activation. Nature 373, 444–448 (1995). 7530337 10.1038/373444a0

[b31] Matsumura R. *et al.* Glandular and extraglandular expression of the Fas-Fas ligand and apoptosis in patients with Sjögren's syndrome. Clin Exp Rheumatol 16, 561–568 (1998). 9779303

[b32] Poulaki V., Mitsiades C. S. & Mitsiades N. The role of Fas and FasL as mediators of anticancer chemotherapy. Drug Resist Updat 4, 233–242 (2001). 11991678 10.1054/drup.2001.0210

[b33] Dharma S. J., Kelkar R. L. & Nandedkar T. D. Fas and Fas ligand protein and mRNA in normal and atretic mouse ovarian follicles. Reproduction 126, 783–789 (2003). 14748697 10.1530/rep.0.1260783

[b34] Rubio Pomar F. J. *et al.* Role of Fas-mediated apoptosis and follicle-stimulating hormone on the developmental capacity of bovine cumulus oocyte complexes *in vitro*. Biol Reprod 71, 790–796 (2004). 15128594 10.1095/biolreprod.104.028613

[b35] Li H. J. *et al.* FasL-induced apoptosis in bovine oocytes via the Bax signal. Theriogenology 80, 248–255 (2013). 23755802 10.1016/j.theriogenology.2013.04.002

[b36] Takahashi T. *et al.* Generalized lymphoproliferative disease in mice, caused by a point mutation in the Fas ligand. Cell 76, 969–976 (1994). 7511063 10.1016/0092-8674(94)90375-1

[b37] Moniruzzaman M., Sakamaki K., Akazawa Y. & Miyano T. Oocyte growth and follicular development in KIT-deficient Fas-knockout mice. Reproduction 133, 117–125 (2007). 17244738 10.1530/REP-06-0161

[b38] Xu J. P., Li X., Mori E., Guo M. W. & Mori T. Aberrant expression and dysfunction of Fas antigen in MRL/MpJ-lpr/lpr murine ovary. Zygote 6, 359–367 (1998). 9921647 10.1017/s096719949800032x

[b39] Guo M. *et al.* Induction of apoptosis mediated by fas receptor and activation of caspase-3 in MRL-+/+ or MRL-lpr/lpr murine oocytes. Zygote 10, 17–22 (2002). 11964087 10.1017/s0967199402002034

[b40] Kubiak J. Z. Mouse oocytes gradually develop the capacity for activation during the metaphase II arrest. Dev Biol 136, 537–545 (1989). 2583375 10.1016/0012-1606(89)90279-0

[b41] Lan G. C. *et al.* Effects of posttreatment with 6-dimethylaminopurine (6-DMAP) on ethanol activation of mouse oocytes at different ages. J Exp Zool A Comp Exp Biol 301, 837–843 (2004). 15449342 10.1002/jez.a.62

[b42] Gordo A. C. *et al.* Intracellular calcium oscillations signal apoptosis rather than activation in *in vitro* aged mouse eggs. Biol Reprod 66, 1828–1837 (2002). 12021069 10.1095/biolreprod66.6.1828

[b43] Guo M. W., Mori E., Xu J. P. & Mori T. Identification of Fas antigen associated with apoptotic cell death in murine ovary. Biochem Biophys Res Commun 203, 1438–1446 (1994). 7524484 10.1006/bbrc.1994.2346

[b44] Guo M. W. *et al.* Expression of Fas ligand in murine ovary. Am J Reprod Immunol 37, 391–398 (1997). 9196798 10.1111/j.1600-0897.1997.tb00249.x

[b45] Mori T. *et al.* Expression of Fas-Fas ligand system associated with atresia through apoptosis in murine ovary. Horm Res **48 Suppl** 3, 11–9 (1997). 9267811 10.1159/000191295

[b46] Serrao K. L., Fortenberry J. D., Owens M. L., Harris F. L. & Brown L. A. Neutrophils induce apoptosis of lung epithelial cells via release of soluble Fas ligand. Am J Physiol Lung Cell Mol Physiol 280, L298–305 (2001). 11159009 10.1152/ajplung.2001.280.2.L298

[b47] Zhang F. *et al.* Phospholipase A2-activating protein (PLAA) enhances cisplatin-induced apoptosis in HeLa cells. Cell Signal 21, 1085–1099 (2009). 19258036 10.1016/j.cellsig.2009.02.018

[b48] Denning T. L. *et al.* Oxidative stress induces the expression of Fas and Fas ligand and apoptosis in murine intestinal epithelial cells. Free Radic Biol Med 33, 1641–1650 (2002). 12488132 10.1016/s0891-5849(02)01141-3

[b49] Vogt M., Bauer M. K., Ferrari D. & Schulze-Osthoff K. Oxidative stress and hypoxia/reoxygenation trigger CD95 (APO-1/Fas) ligand expression in microglial cells. FEBS Lett 429, 67–72 (1998). 9657385 10.1016/s0014-5793(98)00562-6

[b50] MacRedmond R., Singhera G. K. & Dorscheid D. R. Erythropoietin inhibits respiratory epithelial cell apoptosis in a model of acute lung injury. Eur Respir J 33, 1403–1414 (2009). 19164355 10.1183/09031936.00084608

[b51] Ma W. *et al.* Reduced expression of MAD2, BCL2, and MAP kinase activity in pig oocytes after *in vitro* aging are associated with defects in sister chromatid segregation during meiosis II and embryo fragmentation after activation. Biol Reprod 72, 373–383 (2005). 15469999 10.1095/biolreprod.104.030999

[b52] Takahashi T. *et al.* Poor embryo development in mouse oocytes aged *in vitro* is associated with impaired calcium homeostasis. Biol Reprod 80, 493–502 (2009). 19038861 10.1095/biolreprod.108.072017

[b53] Gordo A. C., Wu H., He C. L. & Fissore R. A. Injection of sperm cytosolic factor into mouse metaphase II oocytes induces different developmental fates according to the frequency of [Ca(2t)](i) oscillations and oocyte age. Biol Reprod 62, 1370–1379 (2000). 10775189 10.1095/biolreprod62.5.1370

[b54] Mariani S. M., Matiba B., Bäumler C. & Krammer P. H. Regulation of cell surface APO-1/Fas (CD95) ligand expression by metalloproteases. Eur J Immunol 25, 2303–2307 (1995). 7545118 10.1002/eji.1830250828

[b55] Mariani S. M., Matiba B., Sparna T. & Krammer P. H. Expression of biologically active mouse and human CD95/APO-1/Fas ligand in the baculovirus system. J Immunol Methods 193, 63–70 (1996). 8690931 10.1016/0022-1759(96)00051-8

[b56] Schneider P. *et al.* Conversion of membrane-bound Fas(CD95) ligand to its soluble form is associated with downregulation of its proapoptotic activity and loss of liver toxicity. J Exp Med 187, 1205–1213 (1998). 9547332 10.1084/jem.187.8.1205 PMC2212219

[b57] Rensing-Ehl A. *et al.* Local Fas/APO-1 (CD95) ligand-mediated tumor cell killing *in vivo*. Eur J Immunol 25, 2253–2258 (1995). 7545115 10.1002/eji.1830250821

[b58] Manabe N. *et al.* Role of cell death ligand and receptor system on regulation of follicular atresia in pig ovaries. Reprod Domest Anim **43 Suppl** 2, 268–272 (2008). 18638134 10.1111/j.1439-0531.2008.01172.x

[b59] Jääskeläinen M. *et al.* TRAIL pathway components and their putative role in granulosa cell apoptosis in the human ovary. Differentiation 77, 369–376 (2009). 19281785 10.1016/j.diff.2008.12.001

[b60] Chang A. S., Dale A. N. & Moley K. H. Maternal diabetes adversely affects preovulatory oocyte maturation, development, and granulosa cell apoptosis. Endocrinology 146, 2445–2453 (2005). 15718275 10.1210/en.2004-1472

